# Neurobiology and yoga for managing anxiety and body image in eating disorders-2 case study presentations

**DOI:** 10.1186/2050-2974-3-S1-O17

**Published:** 2015-11-23

**Authors:** Virginia Woods

**Affiliations:** 1 Private Practice, Australia

## 

It is proposed that in clinical practice body oriented exercises can support lowering of activation in the nervous system to allow access to more rational thinking. Activities such as Yoga, exercise, Pilates, Tai Chi, martial arts, dance, movement improvisation can teach clients, to lower the experience of anxiety around body perception and body discomfort, and through the body access resources for anxiety and depression management. This short paper will present research in neurobiology providing an underlying theoretical base, and discuss two case studies that indicate the possibility of yoga and movement activities in clinical settings to provide practical, accessible resources for people with eating disorders to:

• Calm agitation

• Reduce anxious thoughts

• Become present through mindfulness and grounding

• Experience core strength and control over their body

• Challenging body dysmorphia

• Increasing sense of self

• Increase interpersonal skills

• Learn how to slow down and experience deep rest.

Virginia Woods is a registered psychologist in private practice for over 20 years, working with eating disorders for 11 years; and is also a dance-movement & somatic psychotherapist for over 30 years.

